# Record high parainfluenza season in children after relaxation of COVID‐19 restrictions in fall 2021—A nationwide register study in Finland

**DOI:** 10.1111/irv.12983

**Published:** 2022-03-21

**Authors:** Ilari Kuitunen, Miia Artama, Marjut Haapanen, Marjo Renko

**Affiliations:** ^1^ Institute of Clinical Medicine and Department of Pediatrics University of Eastern Finland Kuopio Finland; ^2^ Department of Pediatrics Mikkeli Central Hospital Mikkeli Finland; ^3^ Faculty of Social Sciences Tampere University Tampere Finland; ^4^ Department of Health Security Finnish Institute for Health and Welfare Helsinki Finland; ^5^ Department of Pediatrics Kuopio University Hospital Kuopio Finland

**Keywords:** adenovirus, COVID‐19, influenza, parainfluenza, rhinovirus, RSV

## Abstract

Social restrictions interrupted the circulation of common respiratory viruses among children in spring 2020. In the winter season 2020–2021, only rhinovirus spread in Finland. As the restrictions were ended in September 2021, we saw record high epidemic peak of parainfluenza. Typically, the epidemic peak is in springtime, but now, it started in the early fall 2021. The monthly incidence among children aged 0–4 years (120 per 100,000 children) was six times higher than the second highest reported monthly incidence (21 per 100,000 children) during the last 10 years. Our finding highlights the importance of active surveillance of viral respiratory pathogens during the pandemic.

## 1 BACKGROUND

Social restrictions set against COVID‐19 reduced the circulation of common respiratory pathogens rapidly among children in Finland in spring 2020.^1^, ^2^ This was seen in all common respiratory viruses (influenza A and B, parainfluenza, respiratory syncytial virus [RSV] and rhinovirus),^3^ resulting in lower pediatric emergency department visit rates.^1^, ^4^ As the schools were reopened, rhinovirus started to spread normally.^3^, ^5^ There have been minimal restrictions towards children after May 2020 in Finland, and masking has not been promoted for children aged less than 12 years at any point. Despite the relaxed restrictions, Finland did not have RSV or influenza season in winter 2020–2021 at all,^6^, ^7^ although rhinovirus spread normally.^5^ Pediatric emergency department visit rates returned to normal level already during the summer 2020.^4^

As the last restrictions were abandoned in fall 2021, we saw an increasing trend in the emergency department visits and hospitalizations due to respiratory symptoms in the youngest children (age 0–4 years). Timing of this early epidemic was prior to traditional RSV season and rhinovirus detections remained in normal levels.^8^ Based on the preliminary infectious diseases' surveillance, parainfluenza seemed to be overrepresented in fall 2021.^8^ Previous studies from the USA noted that parainfluenza detections turned to increase in spring 2021 after being in record low levels in 2020.^9^ Therefore, we decided to examine the epidemiology of parainfluenza virus infections in Finnish children during the last 10 years and focused especially to the pandemic era.

## 2 MATERIALS

We conducted a nationwide register‐based retrospective epidemiologic surveillance study. We included all laboratory confirmed parainfluenza virus findings from the National Infectious Diseases Register from January 2012 to December 2021. We included children aged 0–14 and stratified them into three groups (0–4, 5–9, and 10–14 years old). We calculated weekly and monthly incidences per 100,000 children in each age group.

The National Infectious Diseases Register contains all microbiological findings, and all laboratories in Finland are mandated by the law of contagious diseases to report findings of notifiable diseases immediately to the register, which is maintained by the Finnish Institute of Health and Welfare. The coverage of the register is excellent.^8^ Due to the fully register‐based study design, our study did not need ethical committee evaluation or study permission.

## 3 RESULTS

**FIGURE 1:**
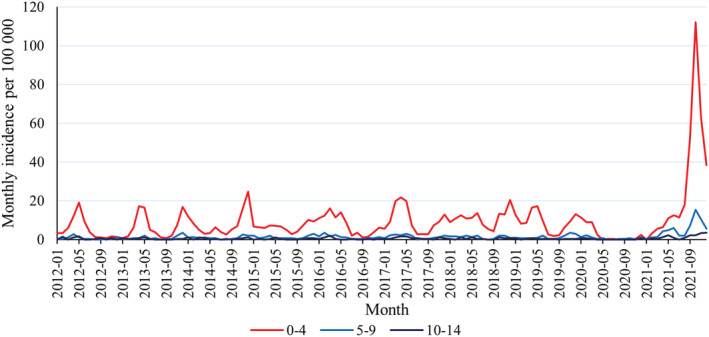
Monthly incidences of laboratory confirmed parainfluenza virus findings per 100,000 children stratified by age (0–4, 5–9, and 10–14 years) in Finland from January 2012 to December 2021

**FIGURE 2:**
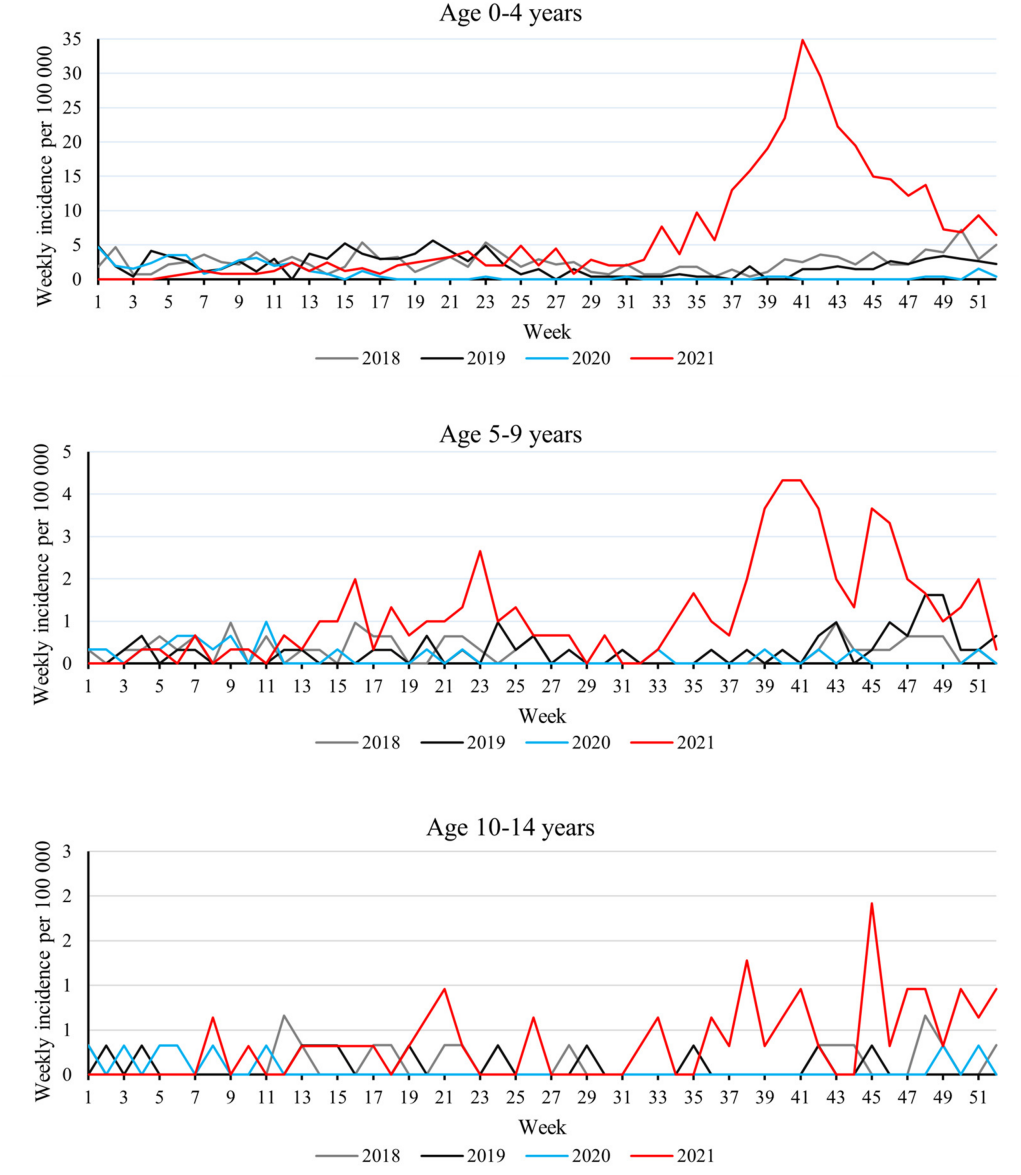
Weekly incidences of parainfluenza virus findings per 100,000 children stratified by age (0–4, 5–9, and 10–14 years) in Finland from 2018 to 2021

We included a total of 3,887 parainfluenza virus detections during our study period (2012–2021), and majority of these (81%) were detected in the youngest age group (age 0–4 years). In the monthly comparison, the incidences remained relatively stable between years until the beginning of the pandemic in March 2020 (Figure 1). Typically, the incidence peaks have been in the spring. During the year 2020, the weekly incidences remained near to zero until the detections were noted from the beginning of year 2021 again. The incidence turned to rapid increase after summer holidays in fall 2021, and this was seen all age groups (Figure 2). The incidence peak was six times higher than any previous peak in the last 10 years (Figure 1). Highest recorded weekly incidence (35 per 100,000) was reported in week 41 of 2021. The peak in 2021 was rapid, and the incidence turned immediately to rapid decline, which has been seen in previous incidence peak as well.

## 4 DISCUSSION

We report a record epidemic of parainfluenza virus detections among Finnish children after the relaxation of social restrictions in fall 2021. The peak occurred in atypical season, as traditionally the parainfluenza peak is observed in the spring. Similar atypical peaks have been reported with rhinoviruses and RSV in Asia, Australia, Europe, and the USA after the relaxation of social restrictions.^10^, ^11^, ^12^, ^13^, ^14^ We did not find any relevant reports outside the USA on the possible parainfluenza resurgence after the restrictions were relaxed.^9^

Our findings highlight the importance of active pathogen surveillance during a pandemic. Majority of the testing resources have been used to test COVID‐19, but we should also recognize other pathogens. In Finland, respiratory viruses are typically tested in pediatric emergency rooms from patients needing inpatient admissions. The testing guideline regarding the use of broader viral sampling has not changed during the COVID‐19 pandemic, although a limitation to our results is the lack of nationwide testing rates as it is not reported to the National Infectious Diseases Register. Furthermore, we lack the hospital data, so we are unable to analyze the clinical spectrum of this record high parainfluenza season. Our main strength is the nationwide register, where all laboratories are mandated by the law of contagious diseases to report all positive findings of notifiable diseases. This allows us to calculate precise nationwide incidence estimates.

In conclusion, we report a record high parainfluenza virus season in Finnish children during the fall 2021. The epidemic peak occurred immediately after the social restrictions were ended in Finland. Our finding highlights the need for active disease surveillance during the pandemic.

## AUTHOR CONTRIBUTIONS

**Ilari Kuitunen:** Conceptualization; formal analysis; investigation; methodology; software; visualization. **Miia Artama:** Conceptualization; data curation; project administration; resources. **Marjut Haapanen:** Investigation; methodology; visualization. **Marjo Renko:** Conceptualization; funding acquisition; project administration; resources; supervision.

### PEER REVIEW

The peer review history for this article is available at https://publons.com/publon/10.1111/irv.12983.

## Data Availability

All data available from the corresponding author per request.
